# Supplementary material for: Pregnancy after Kidney Transplantation—Impact of Functional Renal Reserve, Slope of eGFR before Pregnancy, and Intensity of Immunosuppression on Kidney Function and Maternal Health
Source: J Clin Med. 2023 Feb 15;12(4):1545. doi: 10.3390/jcm12041545 (PMC9964361; doi:10.3390/jcm12041545)
Supplement: Supplementary file 1 [file jcm-12-01545-s001.zip › Supplementary Table S1.pdf]

**Table S1.** Characteristics of allograft failure

| | Main cause of graft loss | Tx | Time gap 1* | Time gap 2** | Mean eGFR (start of observati on) | Pre-existing DSA/ number of MM | Donor age at graft loss | Previous graft rejection | BANFF2017 score from latest graft biopsy | *De novo* DSA (years after start of observation) |
|---|---|---|---|---|---|---|---|---|---|---|
| **Posttransplant pregnancies** | | | | | | | | | | |
| 1 | Severe chronic allograft N | PKTx | 5.6 | 15 | 70 | class II | 35 | Yes (PTx) | g0t0i1v0 cg0ct2ci2ptc0ah3C4D0 | No |
| 2 | Chronic allograft N | KTx | 5 | 15 | 60 | No | 64 | No | g0t0i0v0 cg0ct2ci2ptc0ah2C4D0 | No |
| 3 | Chronic active humoral rejection | KTx | 15.8 | 20 | 64 | No | 36 | No | g1t1i1v0 cg3ct1ci1ptc0ah2c4D0 | 7 (class I) |
| 4 | Chronic humoral rejection | KTx | 17 | 27 | 79 | No | 47 | No | g0t0i0v0 cg3ct3ci3ptc2ah3c4D0 | 19 (class I) |
| 5 | FSGS recurrence | KTx | 3 | 19 | 38 | No | 67 | No | g0t0i0v0 cg0ct1ci1ptc0ah3c4D0 | No |
| 6 | Chronic active humoral rejection | KTx | 12 | 14 | 78 | No | 69 | No | g2t1i2v0 cg3ct1ci1ptc1ah3C4D0 | 13 (class I and II) |
| 7 | Chronic allograft N, fibrosis | KTx | 0.16 | 22 | 24 | NA | 70 | No | g0t0i0v0 cg0ct2ci0ptc0ah3c4D0 | 1.8 (class II) likely as preexisting DSA |

| | | | | | | | | | | |
|---|---|---|---|---|---|---|---|---|---|---|
| **Control cohort** | | | | | | | | | | |
| 1 | Severe chronic allograft N | KTx | 17.5 | 29 | 55 | no | 71 | No | g0t0i0v0<br>cg0ct3ci2ptc0ah3c4D0 | no |
| 2 | Chronic allograft N, death | KTx | 11 | 12 | 42 | no | 67 | No | g0t0i0v0<br>cg0ct1ci1ptc0ah2c4D0 | no |
| 3 | Chronic humoral rejection | KTx | 13 | 14 | 50 | no | 65 | No | g0t0i0v0<br>cg3ct1ci1ptc0 ah3c4D0 | 1 (class II) |
| 4 | ATN, FSGS recurrence (?) | KTx | 2.8 | 8 | 35 | no | 73 | No | g0t0i0v0<br>cg0ct1ci1ah1ptc0c4D0 | 3 (class II) |
| 5 | Chronic active humoral rejection | KTx | 17 | 21 | 42 | no | 70 | Yes | g1t0i1v0<br>cgct0ci1ah2ptc3c4D0 | 0.8 (class II) |

Time gap 1* elapsed time between end of pregnancy/observation until graft loss (years). Time gap 2** elapsed time between date of transplantation until graft loss (years). TX transplantation, PKTX: combined pancreas-kidney transplantation, KTX: kidney transplantation, NA not available; FSGS, ATN=acute tubular necrosis, N=nephropathy. g=glomerulitis, t=tubulitis, i=inflammation, v=vascular, g=chronic glomerulitis, ct=chronic tubulitis, ci=chronic inflammation, ptc=peritubular capillaritis, ah=arterial hyalinosis
