# Supplementary figures and images for: Pregnancy after Kidney Transplantation—Impact of Functional Renal Reserve, Slope of eGFR before Pregnancy, and Intensity of Immunosuppression on Kidney Function and Maternal Health
Source: J Clin Med. 2023 Feb 15;12(4):1545. doi: 10.3390/jcm12041545 (PMC9964361; doi:10.3390/jcm12041545)

## Slide 1
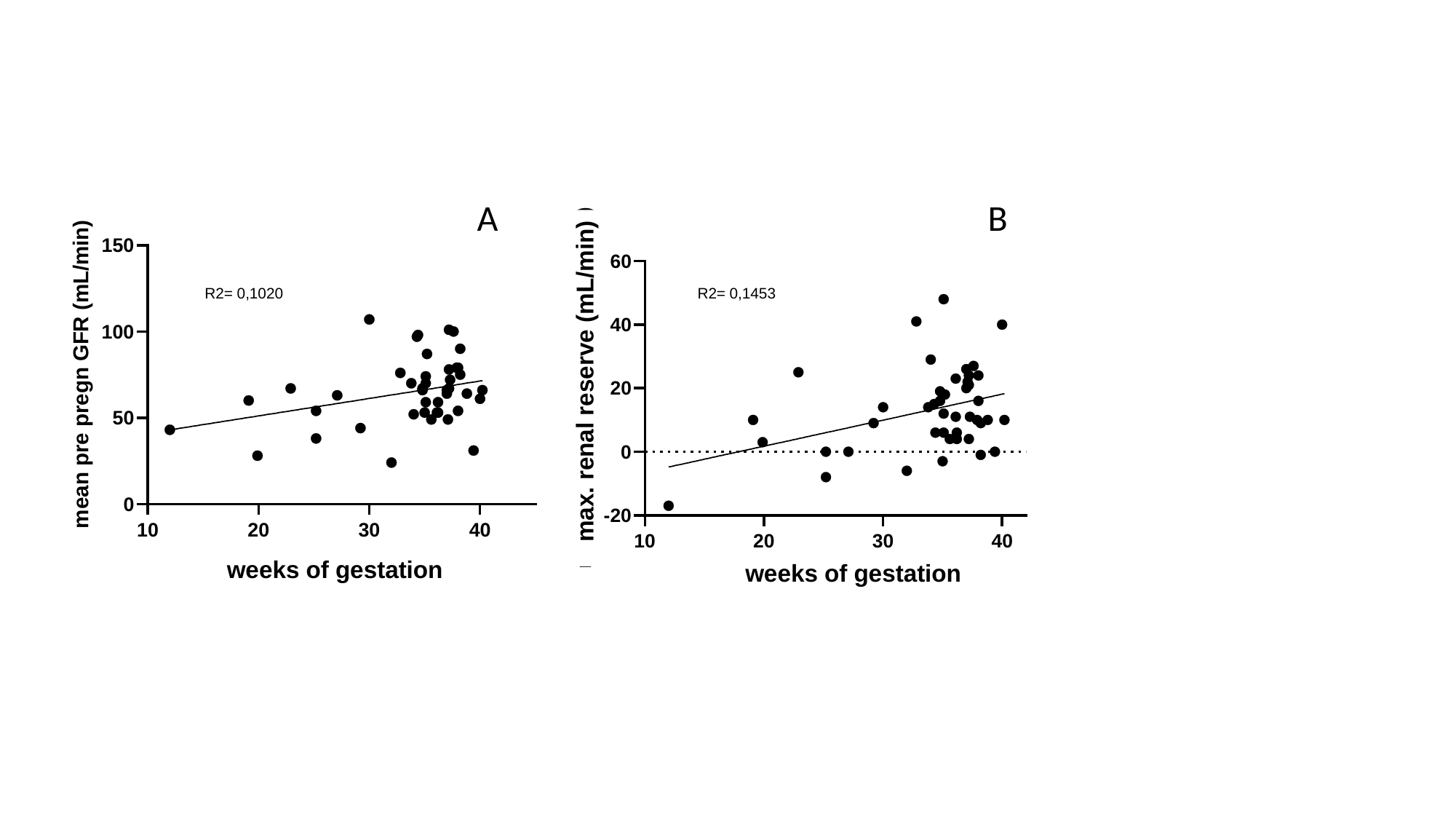

A
B
| R2= 0,1020 |
| --- |
| R2= 0,1453 |
| --- |
 max. renal reserve (mL/min)
weeks of gestation
weeks of gestation

Supplement: Supplementary file 1 [file jcm-12-01545-s001.zip › Supplementary Figure S1.pptx]

## Slide 1
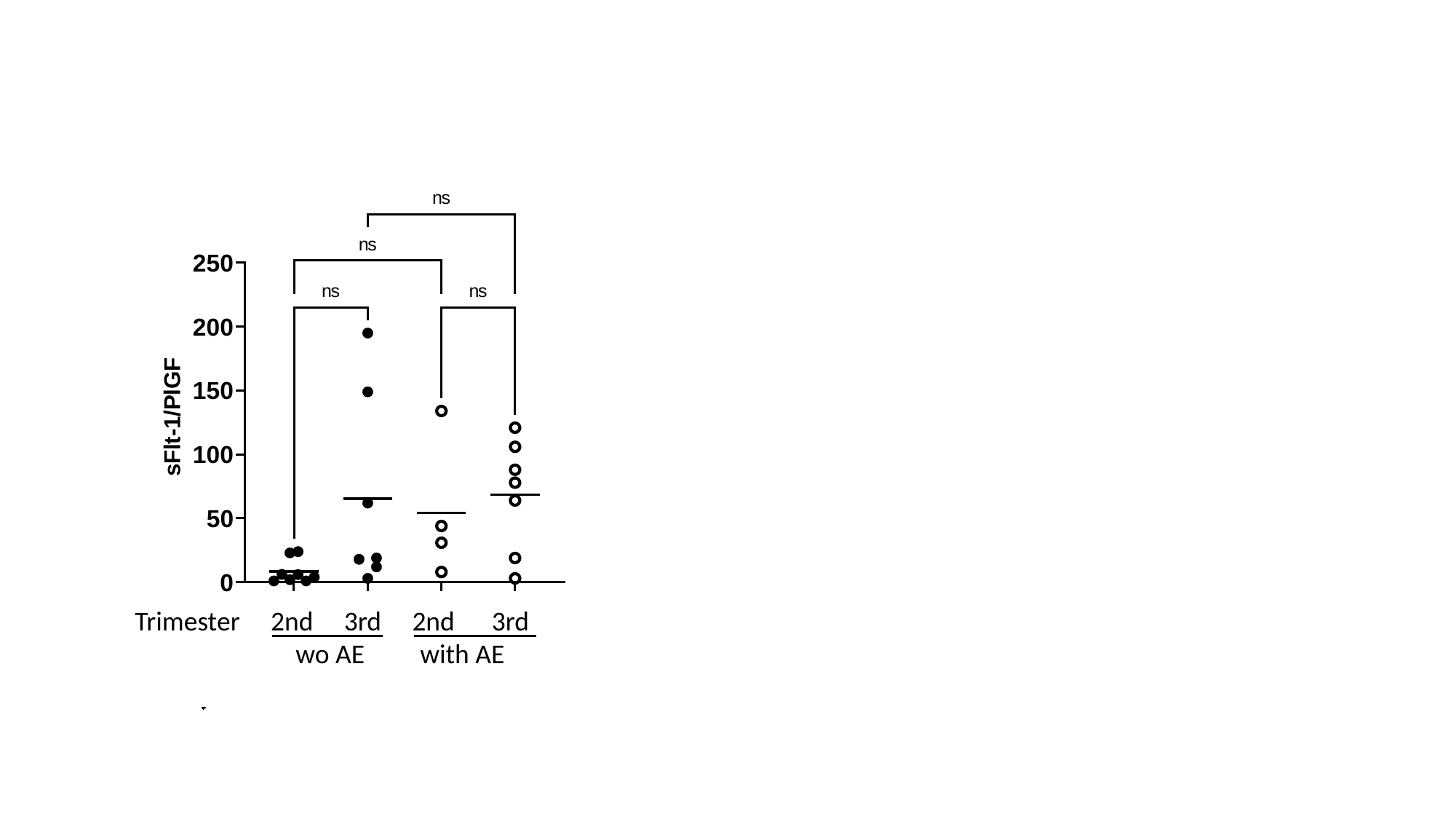

Trimester 2nd 3rd 2nd 3rd
 wo AE with AE

Supplement: Supplementary file 1 [file jcm-12-01545-s001.zip › Supplementary Figure S2.pptx]

## Slide 1
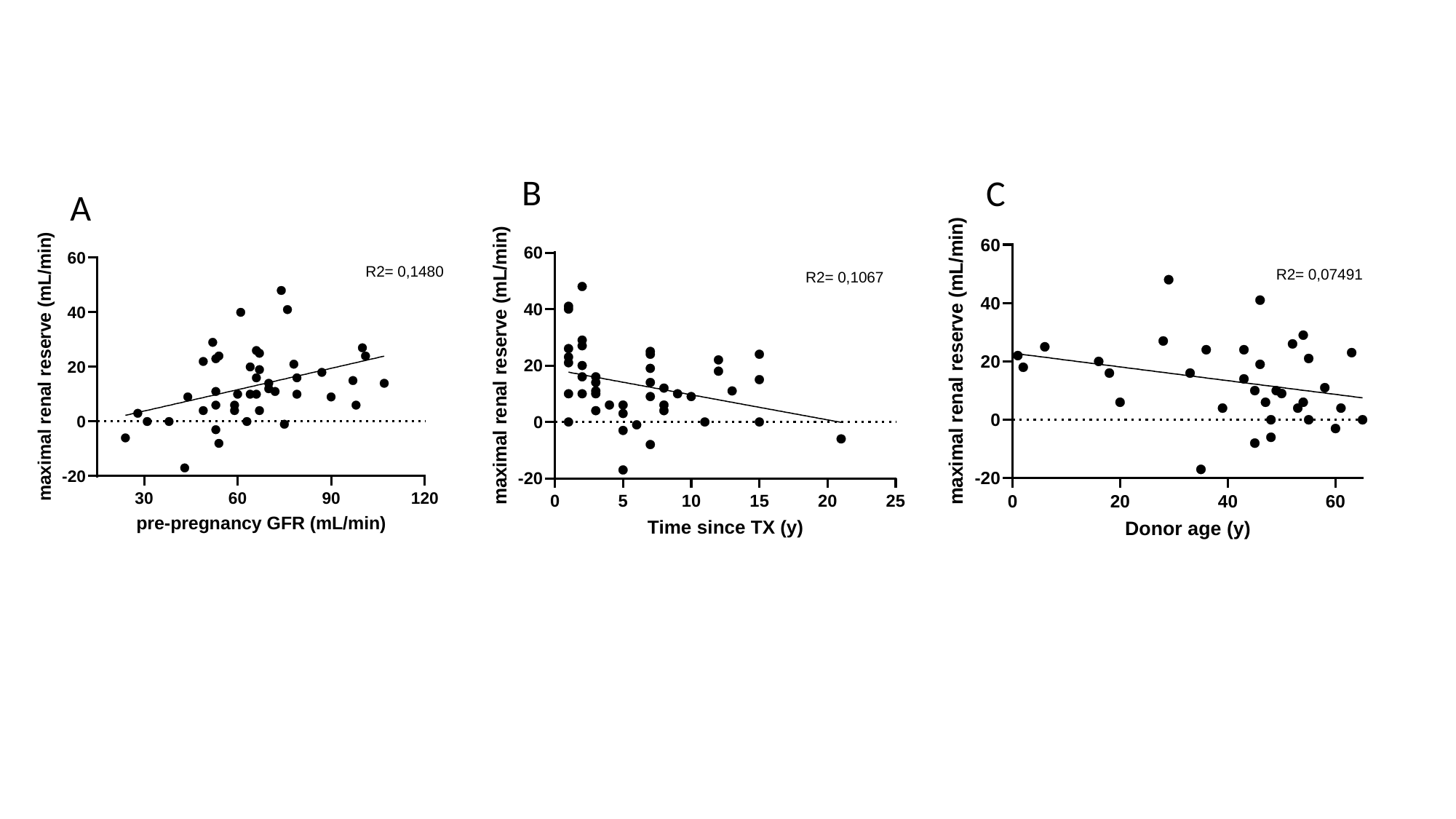

B
C
A
| R2= 0,07491 |
| --- |
| R2= 0,1067 |
| --- |
| R2= 0,1480 |
| --- |

Supplement: Supplementary file 1 [file jcm-12-01545-s001.zip › Supplementary Figure S3.pptx]
